# Case Report: A case report of Moyamoya disease in a 36 year old African American woman

**DOI:** 10.12688/f1000research.5859.1

**Published:** 2014-12-08

**Authors:** Rohit Kumar Gudepu, Mohtashim A. Qureshi, Ihtesham A. Qureshi, Lakshman Rao

**Affiliations:** 1 Dunnerwin Medical Center, Dunn, North Carolina, USA; 2 Mamata Medical College, Khammam, Telangana, India

## Abstract

Moyamoya is a rare idiopathic progressive vaso-occlusive disease characterized by irreversible condition of main blood vessels to the brain as they enter into the skull. We present a case of 36 year old African American female presenting to the Out Patient Clinic with headache which were on and off for 4-6 months and did not relieve on routine medical therapy. It was associated with weakness on right side for last few days. The patient was investigated with CT Angiogram, diagnosed as Moyamoya disease and operated. She has been followed up for the last 5 years and the patient has not complained of any headaches or focal neurological symptoms.

## Introduction

Moyamoya disease is a progressive occlusive condition involving cerebral vessels which includes mainly, stenosis of distal internal carotid arteries on both sides and anterior and middle cerebral arteries thereby leading to development of collateral vessels to compensate for the occlusion. Moyamoya is a rare disease with reported incidence of 0.086 per 100,000 population ^1^. Though, originally considered to affect predominantly persons of Asian heritage it is now seen throughout the world in people of many ethnic backgrounds ^2, 3^. There are nearly twice as many female patients as male patients ^4, 9, 10^. Moyamoya disease usually is associated with condition like Sickle cell disease, Down’s Syndrome and Neurofibromatosis-1 ^4– 7^.

## Case description

A 36 year old African American female presented to the Out Patient Office with headache which was on and off for last 4–6 months. The headache presented was of piercing type with moderate intensity not relieved by butalbital, acetaminophen. It was associated with weakness on right hand side for last few days, which evolved into dizziness and speech difficulty. There was no associated fever, night sweats, loss of consciousness, vomiting, ataxia, photophobia, tingling, numbness, difficulty swallowing, difficulty hearing, neck stiffness, vision problems. Her family history was significant for hypertension, diabetes and coronary artery disease. General physical examination showed no abnormalities. She is obese with BMI of 36.4; Vitals include [Heart Rate = 93/min, Blood Pressure = 165/75 mm Hg, Temp. = 36.67°C, Resp. Rate = 18/min]. Neurological examination showed intact 2–12 cranial nerve function, motor strength grossly intact with strength 3/5 on the right side of hand. No pronator drift, normal muscular tone, and gait was normal. Laboratory investigations, complete blood count, electroencephalogram and echocardiogram were normal.

**Figure 1.**
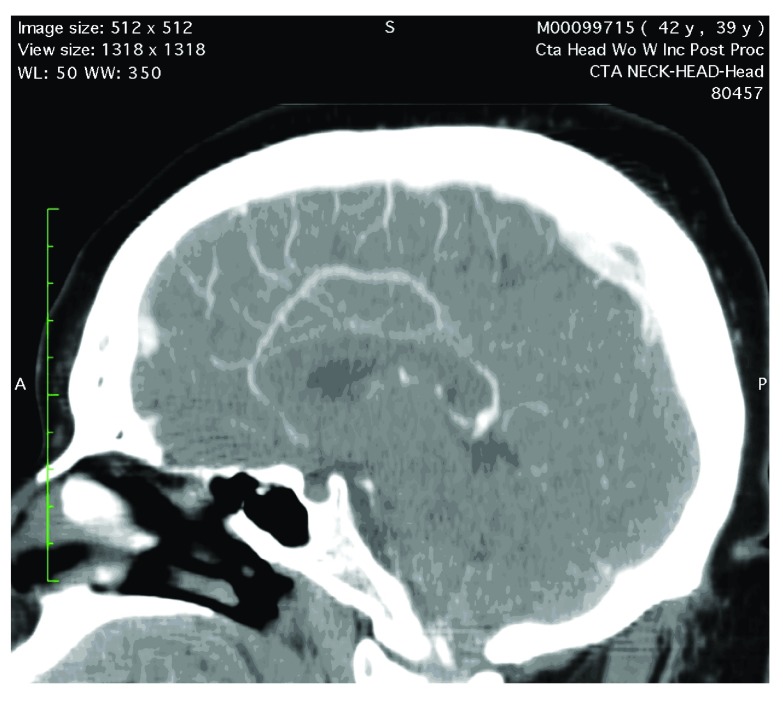
Sagittal view of a CT Angiogram of the head at the level of lateral ventricle demonstrates mild proliferation of the collateral vessels extending towards the vertex.

**Figure 2.**
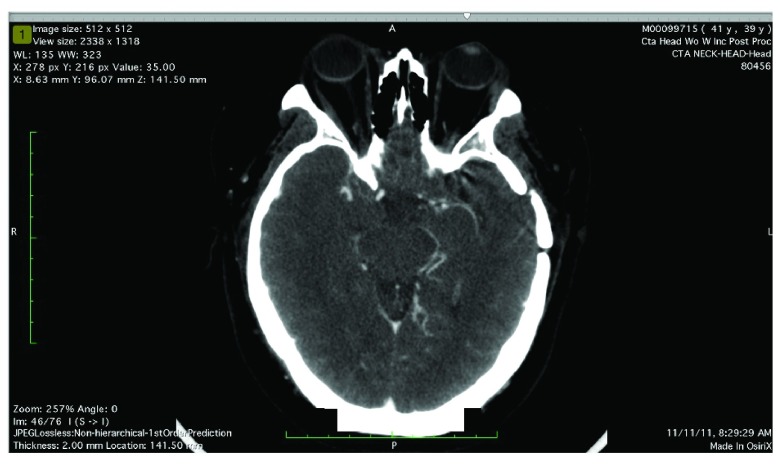
Axial view of CT Angiogram of the head at the level of the Circle of Willis demonstrates mild proliferation of collateral vessels emanating from the distal internal carotid artery, particularly on the left side. In addition, the visualized portion of the distal internal carotid artery and M1 segment of the left middle cerebral artery appear somewhat diminutive in caliber.

**Figure 3.**
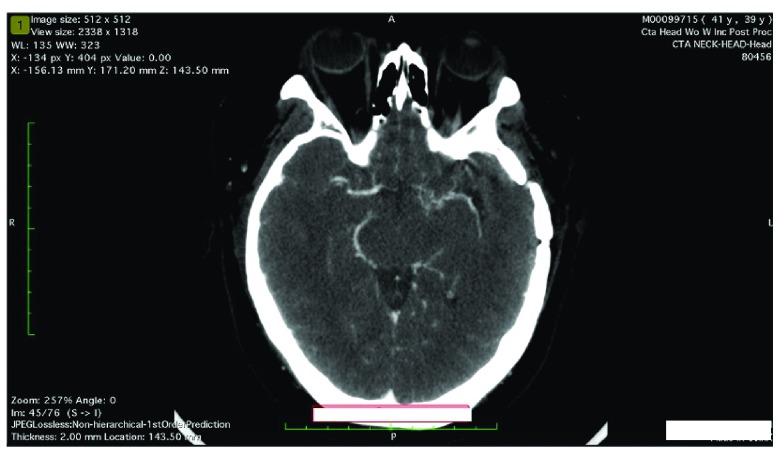
Axial view of CT Angiogram of the head at the level of the Circle of Willis demonstrates mild proliferation of collateral vessels emanating from the distal internal carotid artery, particularly on the left side. In addition, the visualized portion of the distal internal carotid artery and M1 segment of the left middle cerebral artery appear somewhat diminutive in caliber.

A CT angiogram head with contrast shows one sagittal [ Figure 1] and two axial views [ Figure 2, Figure 3] at the level of the lateral ventricle and Circle of Willis, demonstrate mild proliferation of collateral vessels emanating from the distal internal carotid artery particularly on the left side. In addition, the visualized portion of the distal internal carotid artery and M1 segment of the left middle cerebral arteries appeared somewhat diminutive in caliber. Findings were generally compatible with progressive vascular occlusive process which can be seen in Moyamoya disease.

Since the patient was refractory to the pain medications, surgical revascularization was done and the procedure involved left superficial temporal artery-left middle cerebral artery bypass without any complications. Following surgery, she was prescribed 81mg aspirin and discharged home and was followed up initially after surgery once every three months for one year followed by every 6 months for next four years. She did not complain of any headaches or focal neurological symptoms.

## Discussion

Moyamoya disease is a chronic, progressive occlusion of the Circle of Willis arteries that leads to the development of characteristic collateral vessels seen on imaging, particularly cerebral angiography ^8^. Moyamoya was originally considered to affect predominantly persons of Asian heritage but has now been observed throughout the world. The incidence peaks lie within two age groups: children who are 5 years old and adults in their mid 40’s ^9– 12^. Moyamoya disease is rarely seen in the African American population. Uchino *et al.* ^1^ found only 27 African Americans with Moyamoya disease in the states of California and Washington. According to the year 2000 US Census population, only 44 African Americans were diagnosed with Moyamoya every year ^8^.

Moyamoya usually presents with recurrent headaches and is migraine like in quality and refractory to medical therapies. Being a chronic progressive occlusive condition it causes stenosis of intracranial internal carotid arteries and their proximal branches causing reduced blood supply to the anterior surface of brain, thereby leading to the formation of collaterals near the apex of carotids which look like “Puffs of Smoke” known as Moyamoya in Japanese ^13^. The process of blockage, once it begins, tends to continue despite any known medical management unless treated with surgery ^14^.

Though MRI angiography is used to confirm the diagnosis and to see the anatomy of the vessels involved, CT angiography can also be used to see intracranial stenoses suggesting Moyamoya. Thus, CT angiography can be considered when MRI is not readily available and a diagnosis of cerebral occlusive vasculopathy is being considered ^15^. Since surgery is the only viable option, revasularization procedures are gaining importance as a primary treatment for Moyamoya, given the poor response to medical therapy and documented success of surgery ^17^.

A good option for adult symptomatic patients is superficial temporal artery-middle cerebral artery bypass or middle meningeal artery to middle cerebral artery bypass ^18– 21^. Patients have reported to have 96% probability of remaining stroke free over the subsequent five years ^4, 16^. The present patient had a successful surgical treatment without any recurrence of symptoms. Nevertheless, the outcome can be predicted based on the neurological status at the time of treatment, more than the patient’s age ^4^. Hence, it is necessary for early diagnosis of the condition followed by surgical intervention therapy. The estimated rate of symptomatic progression is only 2.6% after surgery according to a meta-analysis involving 1156 patients ^17^.

This case highlights the importance of considering Moyamoya disease as one of the differentials while dealing with patients with recurrent headaches who are in their third/fourth decade of life and children around 5 years old in whom headache is not relieved on routine medical treatment. It also emphasizes on the rare presentation among African American population and use of CT angiography as an alternative diagnostic imaging tool for diagnosing Moyamoya in cases of MRI non-availability.

## Patient consent

Informed written consent for publication of clinical details was obtained from the patient.
