# Supplementary material for: Case Report: A case report of Moyamoya disease in a 36 year old African American woman
Source: F1000Res. 2014 Dec 8;3:297. [Version 1] doi: 10.12688/f1000research.5859.1 (PMC4329669; doi:10.12688/f1000research.5859.1)
Supplement: Peer review history

# Peer review: Referee response for version 1

**DOI:** 10.5256/f1000research.6261.r7591

**Published:** 2015-02-16

**Authors:** Johannes Boltze

**Affiliations:** 1 Department of Cell Therapies, Fraunhofer-Institut für Zelltherapie und Immunologie IZI, Leipzig, Germany

This manuscript delivers a comprehensive, well-structured and interesting case study on Moya-Moya. The paper is nicely focused and compact, also providing a well-balanced discussion.

Apart from agreeing on Dr. Campell’s comment, the only recommendation I have is to avoid repetitive text elements. Particularly, the first few sentences in the second paragraph of the case description are almost identical to the legend of Figure 2 and may therefore require rephrasing.

I have read this submission. I believe that I have an appropriate level of expertise to confirm that it is of an acceptable scientific standard.

# Peer review: Referee response for version 1

**DOI:** 10.5256/f1000research.6261.r6982

**Published:** 2014-12-16

**Authors:** Bruce Campbell

**Affiliations:** 1 Department of Neurology, Royal Melbourne Hospital, Melbourne, VIC, Australia

An interesting rare disease case report - NB in Intro states "Moyamoya disease usually is associated with condition like Sickle cell disease, Down’s Syndrome and Neurofibromatosis" -whilst all of these associations are correct, the majority of Moyamoya patients do not have one of these conditions.

I have read this submission. I believe that I have an appropriate level of expertise to confirm that it is of an acceptable scientific standard.
